# Supplementary material for: Matrix regulation: a plug-and-tune method for combinatorial regulation in Saccharomyces cerevisiae
Source: Nat Commun. 2025 Aug 15;16:7624. doi: 10.1038/s41467-025-62886-5 (PMC12356856; doi:10.1038/s41467-025-62886-5)
Supplement: Supplementary file 1 — Supplementary Information [file 41467_2025_62886_MOESM1_ESM.pdf]

Matrix Regulation: a plug-and-tune method for combinatorial regulation in *Saccharomyces cerevisiae*

Teng et al.

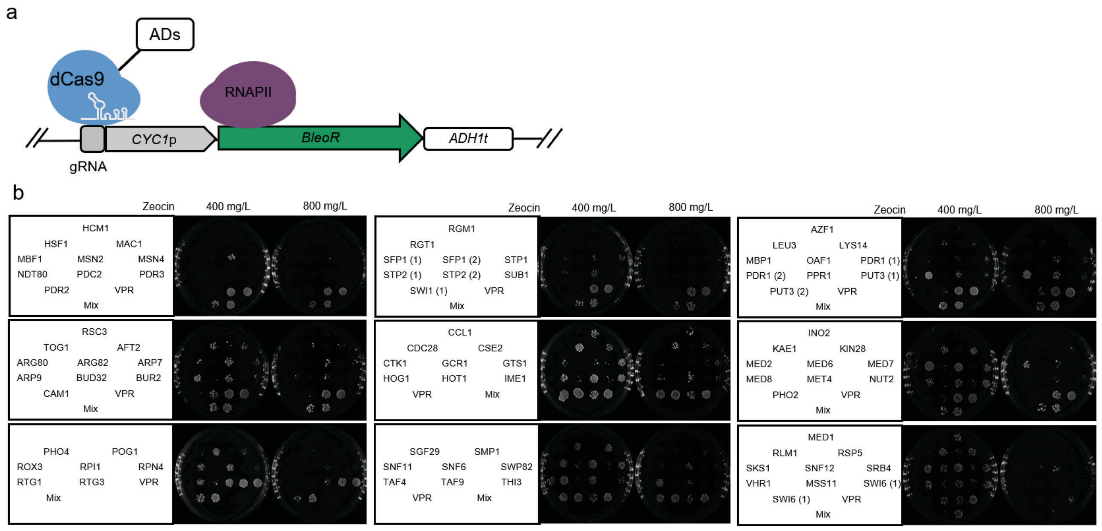

Supplementary Fig. 1 | Characterization of endogenous activators. a The efficiency of endogenous activators was characterized by activating the Zeocin resistance gene ZeoR. ADs represent 111 potential activators. RNAPII indicates RNA polymerase II. **b** Cell counts of the left and the right spot were 0.01 and 0.05 OD_{600}, respectively. The serial dilution was spotted in a plate when the OD_{600} was approximately 1. Mix indicated an equimolar mixture of all strains in the group, with cell count in the spot remaining unchanged. Results show the cell resistance after three days. The upstream regions of ADs containing the DNA-binding domains were designated as (1), and the downstream regions were designated as (2). Potential activators in *ACA1*, *ARO80*, *BAS1*, *CAD1*, *CST6*, *GLN3*, *HAA1*, *HAC1*, *SWI1*, *SWI3*, *THI2*, *UGA3*, *UPC2*, *YAP7*, and *YPR1* showed no growth of any spot except for the controls, so they were not displayed here.

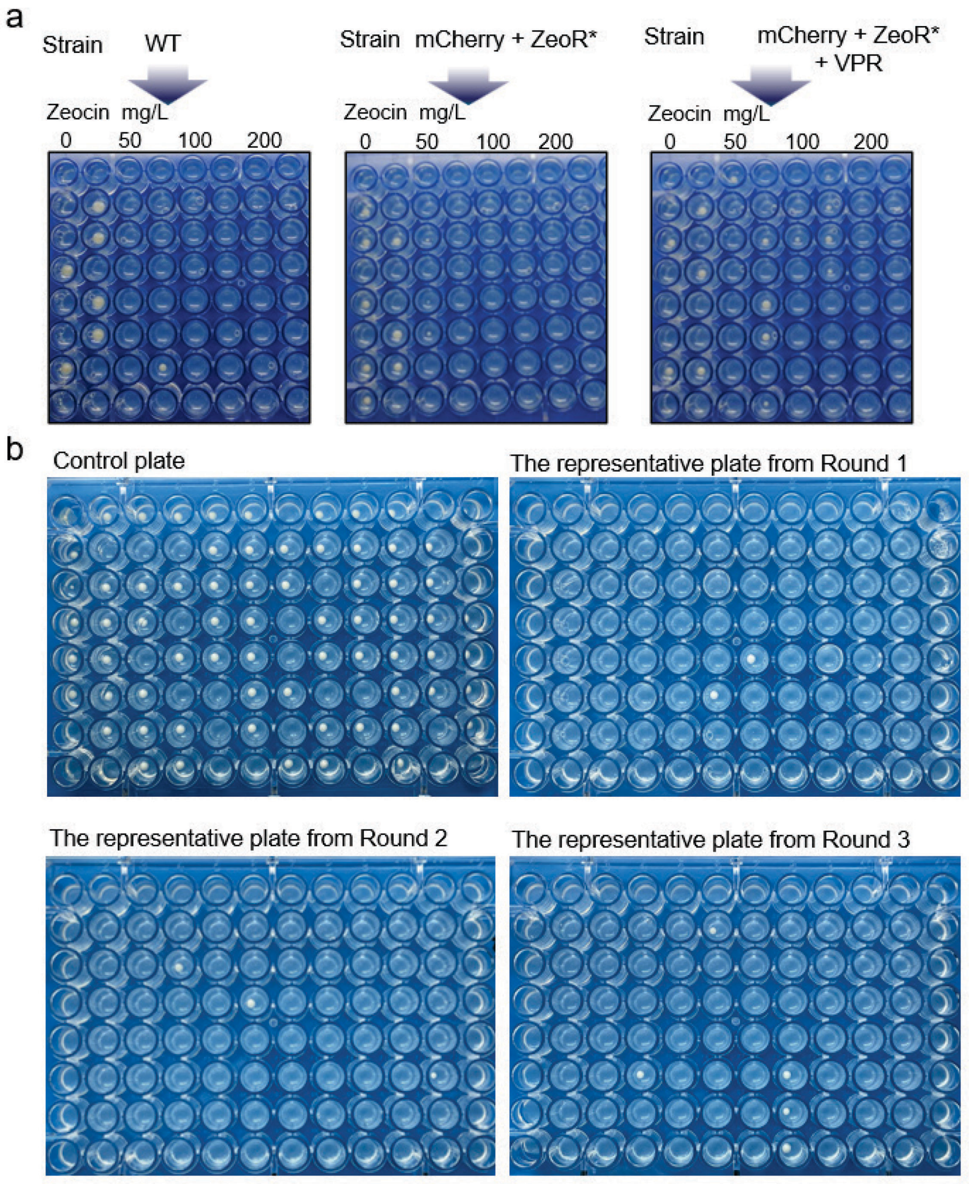

Supplementary Fig. 2 | Original images of 96-well plates. a FACS sorting for increased mCherry and ZeoR expressions. Colonies in the VPR activation group displayed increased mCherry signals and could grow at Zeocin concentrations up to 100 mg/L, while cells without VPR activation grew only at concentrations up to 50 mg/L. **b** The representative plates from 3 rounds of VPR screening. The control plate (without Zeocin) showed a growth rate of ~70%, whereas the Zeocin-containing plates had 2-5 colonies per plate. Images were captured three days after FACS screening.

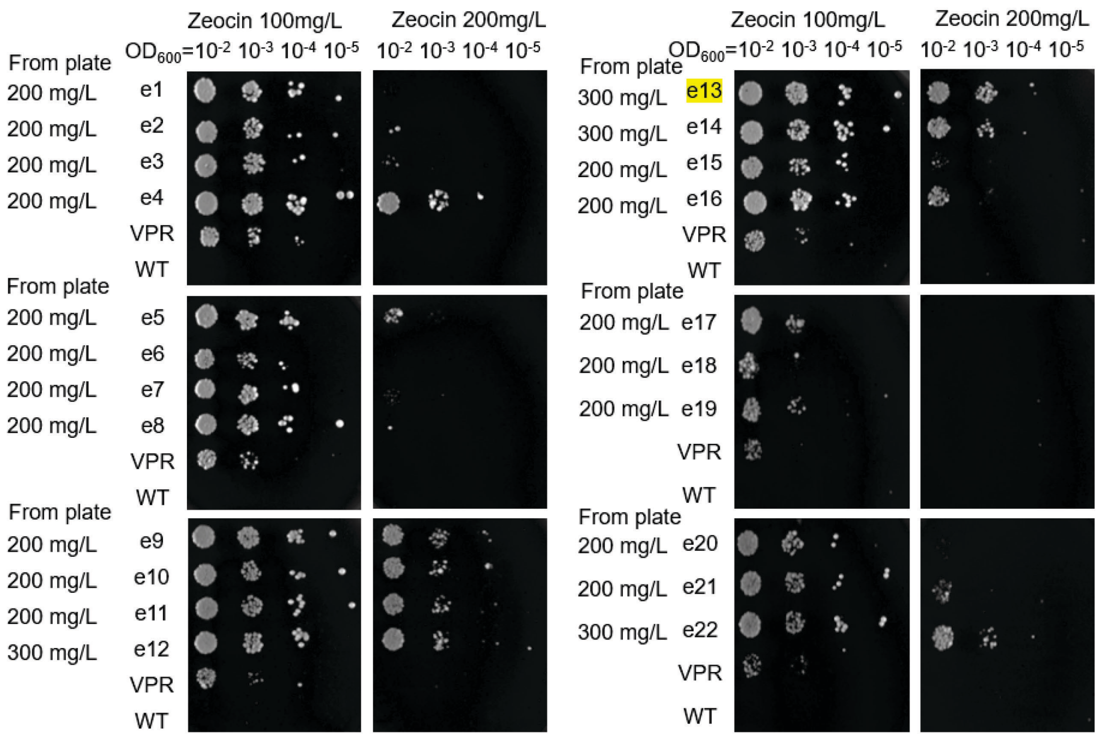

Supplementary Fig. 3 | Characterization of the sorted strains by FACS. e1-e22 represent the strains that were able to grow in 96-well plates after the first round of high-throughput FACS screening. 5D contains only the URA3 plasmid. In this round, strain e13 with a yellow background indicates the optimal mutant strain after screening. Results show the Zeocin resistance of the spots grew after three days.

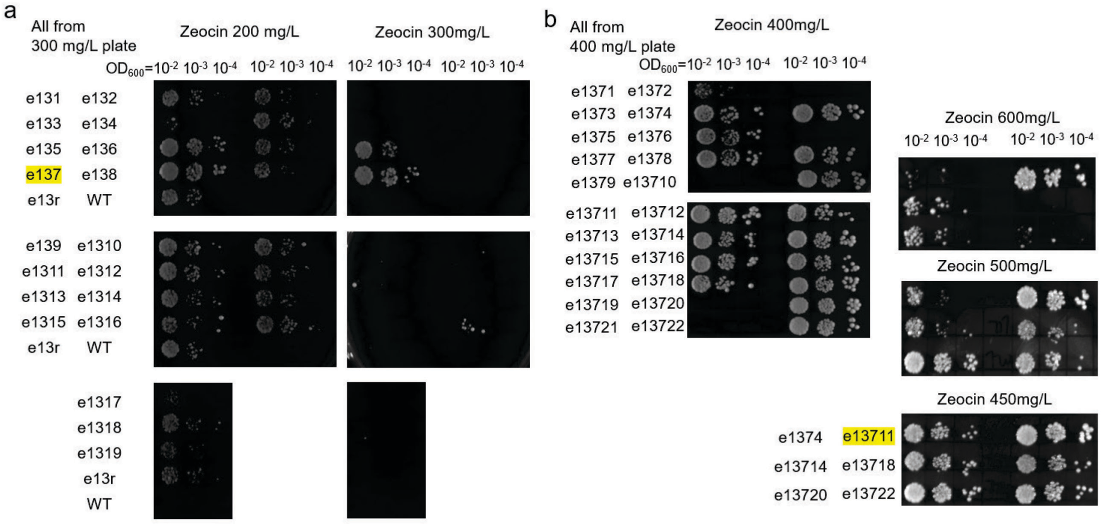

Supplementary Fig. 4 | Characterization of the sorted strains from Round 2 and 3. a e131-e1319 represent the 19 strains that were able to grow in 96-well plates after the second round of high-throughput FACS screening. e13r represents the optimal strain obtained from the first round of screening, with its mutated VPR gene recovered to the original strain. **b** e1371-e13722 represent the strains that were able to grow in 96-well plates after the third round of high-throughput FACS screening. In this round, strain e137 with a yellow background indicates the optimal mutant strain after screening. e137r represents the optimal strain obtained from the second round of screening, with its mutated VPR gene recovered to the original strain. WT contains only the URA3 plasmid. Results show the Zeocin resistance of the spots grew after 3 days.

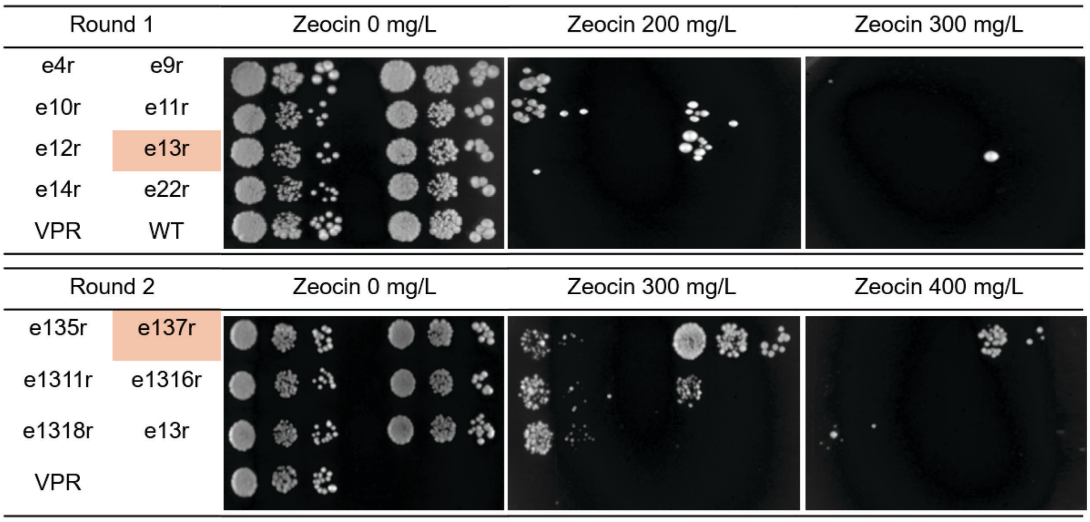

Supplementary Fig. 5 | Spot growth assays of reconstructed VPR strains. The strain numbers correspond to the selected strains in Supplementary Fig. 3 and 4, with r representing reconstruction. The strains e13r and e137r with orange backgrounds indicate the optimal mutant strain after screening.

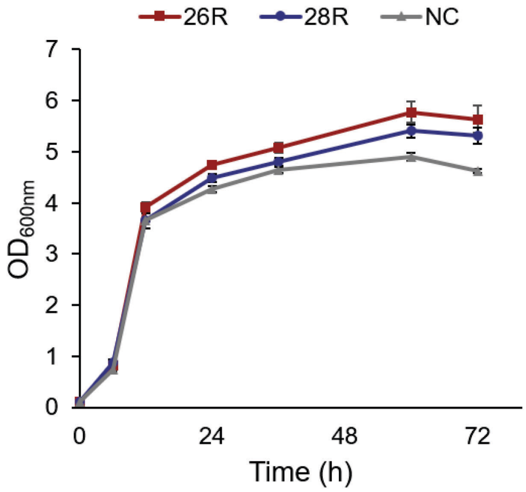

Supplementary Fig. 6 | Growth of the two high squalene producers acquired by MR. The strain without gRNAs was used as the negative control (NC). Error bars represent the standard deviation of three biological replicates (n=3). Source data are provided as a Source Data file.

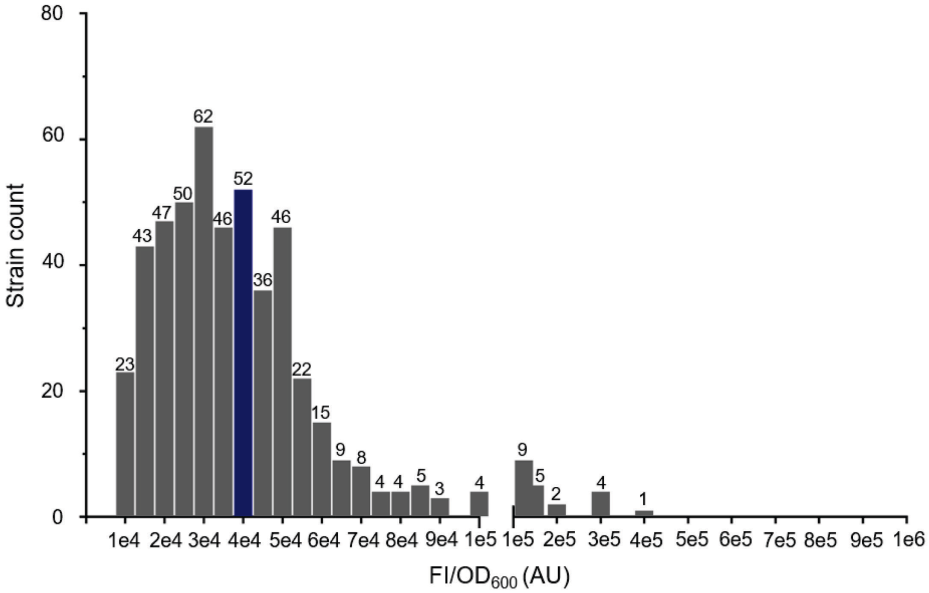

Supplementary Fig. 7 | Semi-high-throughput screening and analysis of 500 strains regulated by the NGG PAM-based MR. The test strains were randomly selected from the SC-His-Ura-Leu drop-out plate with the strain possessing blank plasmid served as the control. Gray bars represent the distribution of test strains, while blue bar indicates the FI range of control. Source data are provided as a Source Data file.

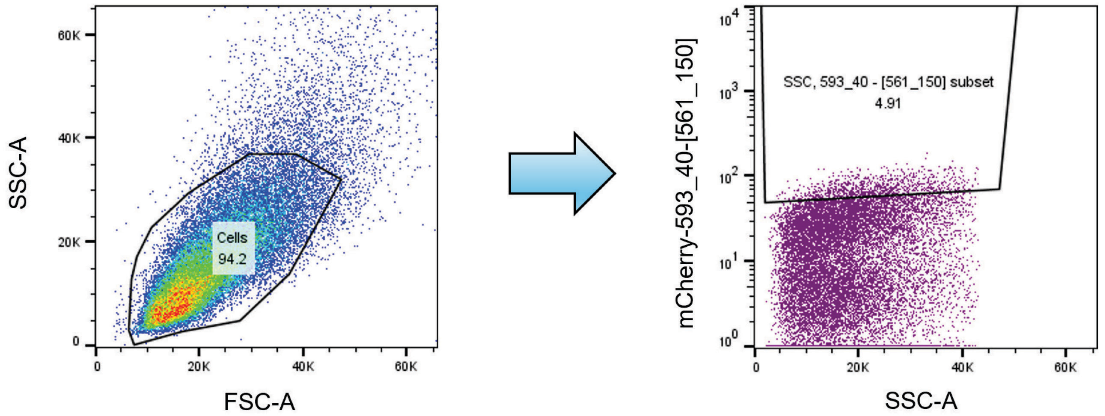

Supplementary Fig. 8 | Example schematic of FACS gating strategy. Firstly, yeast cells were separated from lumps or debris using FSC-A and SSC-A. Then, by plotting mCherry-593_40 - [561_150] and SSC-A, the top 5% of the most fluorescent cells were gated and sorted to 96-well culture plate for further analysis.
