# Supplementary material for: Matrix regulation: a plug-and-tune method for combinatorial regulation in Saccharomyces cerevisiae
Source: Nat Commun. 2025 Aug 15;16:7624. doi: 10.1038/s41467-025-62886-5 (PMC12356856; doi:10.1038/s41467-025-62886-5)
Supplement: Supplementary file 2 — Description of Additional Supplementary Information [file 41467_2025_62886_MOESM2_ESM.pdf]

Description of Additional Supplementary Files

File Name: Supplementary Data 1
Description: Sequences of tRNAs used in this study.

File Name: Supplementary Data 2
Description: Details of endogenous activators.

File Name: Supplementary Data 3
Description: Sequences of VPR and mutants identified in this study.

File Name: Supplementary Data 4
Description: List of strains and plasmids used in this study.

File Name: Supplementary Data 5
Description: List of primers used in this study.

File Name: Supplementary Data 6
Description: List of gRNA 20bp.

File Name: Supplementary Data 7
Description: List of primers used for real-time qPCR.

File Name: Supplementary Data 8
Description: Raw mass spectrometry data used for the analysis of MVA pathway metabolites.
