# Supplementary material for: Matrix regulation: a plug-and-tune method for combinatorial regulation in Saccharomyces cerevisiae
Source: Nat Commun. 2025 Aug 15;16:7624. doi: 10.1038/s41467-025-62886-5 (PMC12356856; doi:10.1038/s41467-025-62886-5)
Supplement: Supplementary file 11 — Reporting Summary [file 41467_2025_62886_MOESM11_ESM.pdf]

nature portfolio

Corresponding author(s): Prof. Dr. Zihe Liu

Last updated by author(s): Jul 26, 2025

# Reporting Summary

Nature Portfolio wishes to improve the reproducibility of the work that we publish. This form provides structure for consistency and transparency in reporting. For further information on Nature Portfolio policies, see our Editorial Policies and the Editorial Policy Checklist.

## Statistics

For all statistical analyses, confirm that the following items are present in the figure legend, table legend, main text, or Methods section.

| n/a | Confirmed | |
|---|---|---|
| ☐ | ☒ | The exact sample size (*n*) for each experimental group/condition, given as a discrete number and unit of measurement |
| ☐ | ☒ | A statement on whether measurements were taken from distinct samples or whether the same sample was measured repeatedly |
| ☐ | ☒ | The statistical test(s) used AND whether they are one- or two-sided<br>*Only common tests should be described solely by name; describe more complex techniques in the Methods section.* |
| ☒ | ☐ | A description of all covariates tested |
| ☒ | ☐ | A description of any assumptions or corrections, such as tests of normality and adjustment for multiple comparisons |
| ☐ | ☒ | A full description of the statistical parameters including central tendency (e.g. means) or other basic estimates (e.g. regression coefficient) AND variation (e.g. standard deviation) or associated estimates of uncertainty (e.g. confidence intervals) |
| ☐ | ☒ | For null hypothesis testing, the test statistic (e.g. *F*, *t*, *r*) with confidence intervals, effect sizes, degrees of freedom and *P* value noted<br>*Give P values as exact values whenever suitable.* |
| ☒ | ☐ | For Bayesian analysis, information on the choice of priors and Markov chain Monte Carlo settings |
| ☒ | ☐ | For hierarchical and complex designs, identification of the appropriate level for tests and full reporting of outcomes |
| ☒ | ☐ | Estimates of effect sizes (e.g. Cohen's *d*, Pearson's *r*), indicating how they were calculated |

*Our web collection on statistics for biologists contains articles on many of the points above.*

## Software and code

Policy information about availability of computer code

| | |
|---|---|
| Data collection | HPLC data were collected using LabSolutions v5.93.<br>Real-time qPCR data were collected using QuantStudio Design&Analysis Desktop Software v1.4.1.<br>Microplate reader data were collected using SoftMax Pro 7.1.<br>FACS data were acollected using BD FACS Software sorter software. |
| Data analysis | HPLC data were analyzed using LabSolutions v5.93.<br>FACS data were analyzed using FlowJo v10.8.1.<br>Real-time qPCR data were analyzed using Prism 10.1.2 software.<br>Mass spectrum data were analyzed using MultiQuant 3.0.3 software.<br>All the other data were exported by Microsoft Excel (365). |

For manuscripts utilizing custom algorithms or software that are central to the research but not yet described in published literature, software must be made available to editors and reviewers. We strongly encourage code deposition in a community repository (e.g. GitHub). See the Nature Portfolio guidelines for submitting code & software for further information.

## Data

Policy information about availability of data

All manuscripts must include a data availability statement. This statement should provide the following information, where applicable:

- Accession codes, unique identifiers, or web links for publicly available datasets
- A description of any restrictions on data availability
- For clinical datasets or third party data, please ensure that the statement adheres to our policy

The mass spectrometry data generated in this study for the analysis of MVA pathway metabolites are provided in Supplementary Data 8. Source data are provided with this paper.

## Research involving human participants, their data, or biological material

Policy information about studies with human participants or human data. See also policy information about sex, gender (identity/presentation), and sexual orientation and race, ethnicity and racism.

| | |
|---|---|
| Reporting on sex and gender | Our study did not involve human participants. |
| Reporting on race, ethnicity, or other socially relevant groupings | Our study did not involve human participants. |
| Population characteristics | Our study did not involve human participants. |
| Recruitment | Our study did not involve human participants. |
| Ethics oversight | Our study did not involve human participants. |

Note that full information on the approval of the study protocol must also be provided in the manuscript.

# Field-specific reporting

Please select the one below that is the best fit for your research. If you are not sure, read the appropriate sections before making your selection.

[x] Life sciences [ ] Behavioural & social sciences [ ] Ecological, evolutionary & environmental sciences

For a reference copy of the document with all sections, see nature.com/documents/nr-reporting-summary-flat.pdf

# Life sciences study design

All studies must disclose on these points even when the disclosure is negative.

| | |
|---|---|
| Sample size | No calculations were performed to determine the sample size. Colonies were selected randomly from agar plates when being prepared for experimental pre-cultures, a single colony represents one biological replicate (sample). Sample sizes were chosen based on common practices in the field and previous studies with similar experimental designs. For tRNA characterization, the experiment was run with six biological replicates. For FI of mCherry, real-time qPCR, and growth assay, each experiment was run with three or four biological replicates. For the verification of the re-transformed strains, the experiment was run with two biological replicates. Sample sizes in flow cytometry were minimally 50, 000. All sample sizes were chosen to allow for statistical analysis and to ensure reproducibility. |
| Data exclusions | No data were excluded. |
| Replication | For tRNA characterization, the experiment was run with six biological replicates. For FI of mCherry, real-time qPCR, and growth assay, each experiment was run with three or four biological replicates. For the verification of the re-transformed strains, the experiment was run with two biological replicates. Sample sizes were chosen based on common practices in the field and previous studies with similar experimental designs. |
| Randomization | Randomization is applicable in this study. Yeast colonies were selected randomly from agar plates when being prepared for experimental pre-cultures. Control/reference samples were included in each group. |
| Blinding | The investigators were blinded to group allocation during data collection and analysis (including HPLC, real-time qPCR, LC-MS, and flow cytometry) of all data presented in the paper. |

# Reporting for specific materials, systems and methods

We require information from authors about some types of materials, experimental systems and methods used in many studies. Here, indicate whether each material, system or method listed is relevant to your study. If you are not sure if a list item applies to your research, read the appropriate section before selecting a response.

## Materials & experimental systems

| n/a | Involved in the study |
|---|---|
| ☒ | ☐ Antibodies |
| ☒ | ☐ Eukaryotic cell lines |
| ☒ | ☐ Palaeontology and archaeology |
| ☒ | ☐ Animals and other organisms |
| ☒ | ☐ Clinical data |
| ☒ | ☐ Dual use research of concern |
| ☒ | ☐ Plants |

## Methods

| n/a | Involved in the study |
|---|---|
| ☒ | ☐ ChIP-seq |
| ☐ | ☒ Flow cytometry |
| ☒ | ☐ MRI-based neuroimaging |

# Plants

| | |
|---|---|
| Seed stocks | Our study did not involve plants. |
| Novel plant genotypes | Our study did not involve plants. |
| Authentication | Our study did not involve plants. |

# Flow Cytometry

## Plots

Confirm that:

☒ The axis labels state the marker and fluorochrome used (e.g. CD4-FITC).

☒ The axis scales are clearly visible. Include numbers along axes only for bottom left plot of group (a 'group' is an analysis of identical markers).

☐ All plots are contour plots with outliers or pseudocolor plots.

☒ A numerical value for number of cells or percentage (with statistics) is provided.

## Methodology

| | |
|---|---|
| Sample preparation | After transformation, each 1 mL of yeast cells was plated onto a SC-URA plate and incubated for three days. The cells were then scraped off using sterile water and cultured overnight. The culture was transferred to a fresh medium and diluted to an OD 600nm of 0.1, then cultured for three days in preparation for FACS. |
| Instrument | BD Influx Cell Sorter |
| Software | BD FACS Software for data collection and FolwJo_V10.8.1 for data analysis. |
| Cell population abundance | The top 5% or 1% most fluorescent cells were collected into 96-well cell culture plates. |
| Gating strategy | Cells were firstly gated on FSC-A and SSC-A for exclusion of dead cells or cell lumps. |

☒ Tick this box to confirm that a figure exemplifying the gating strategy is provided in the Supplementary Information.
